# Convening Partners: Lessons for Sustaining State-University & Community Partnerships in Aging & Behavioral Health

**DOI:** 10.1093/geroni/igaf122.1981

**Published:** 2025-12-31

**Authors:** Paula Carder, Karen Cellarius, Lindsey Smith, Allyson Stodola, Walter Dawson, Leah Brandis

**Affiliations:** Portland State University, Portland, Oregon, United States; Portland State University, Portland, Oregon, United States; Oregon Health & Science University, Portland, Oregon, United States; Portland State University, Portland, Oregon, United States; Oregon Health & Science University, Portland, Oregon, United States; Oregon Health & Science University, Portland, Oregon, United States

## Abstract

OCEBHA is a state-university-community agency partnership in Oregon, a state with a long history of policy successes driven by advocates, policy entrepreneurs, public agency employees and community organizations. These efforts have resulted in innovative home and community-based services for older adults, and an expansion of non-institutional Medicaid-funded and private pay services. We describe how this tradition of partnerships advanced older adult behavioral health on the state policy agenda over time. In 2015, a workgroup composed of aging services and mental health agencies, health care providers, quality improvement, and advocacy organizations identified gaps in and recommended changes to the behavioral health and aging arena, resulting in the establishment of the Older Adult Behavioral Health Initiative (OABHI). The success of this initiative, which included timely access to care from qualified providers who collaborate to provide coordinated, quality, and culturally responsive BH and wellness services, established OCEBHA’s foundation. The organizational structure includes multiple state, university, and community organizations, a national advisory council with experts in workforce, aging, mental health, substance use, and public policy, and a local steering committee including individuals with lived experience. We describe how these partnerships were created and supported, including setting expectations, sharing values and defining goals. In addition, we provide examples of tools, agreements and planning processes that others may use to create and sustain state-university-community partnerships that advance the health and well-being of older adults, especially those with behavioral health needs.

